# Bone-Anchored Hearing Aid vs. Reconstruction of the External Auditory Canal in Children and Adolescents with Congenital Aural Atresia: A Comparison Study of Outcomes

**DOI:** 10.3389/fped.2014.00005

**Published:** 2014-01-22

**Authors:** Soroush Farnoosh, F. Tania Mitsinikos, Dennis Maceri, Debra M. Don

**Affiliations:** ^1^School of Medicine and Biomedical Sciences, The State University of New York at Buffalo, Buffalo, NY, USA; ^2^Keck School of Medicine, University of Southern California, Los Angeles, CA, USA; ^3^Department of Otolaryngology – Head and Neck Surgery, Children’s Hospital Los Angeles, Los Angeles, CA, USA

**Keywords:** BAHA, congenital aural atresia, external auditory canal reconstruction, bone-anchored hearing aid, ear canal

## Abstract

**Objectives/hypothesis:** Congenital aural atresia is a rare condition affecting 1 in 10,000–20,000 children a year. Surgery is required to restore hearing to facilitate normal development. The objective of this study was to compare outcomes in hearing, complications, and quality of life of surgical reconstruction of the external auditory canal reconstruction (EACR) and bone-anchored hearing aid (BAHA) in a pediatric population with congenital aural atresia.

**Study design:** Subjects were children who had a diagnosis of congenital aural atresia or stenosis and who received either BAHA or EACR.

**Methods:** The medical records of 68 children were reviewed for operative complications and audiometric results. A quality of life questionnaire was prospectively administered to a subset of subjects.

**Results:** Pre-operatively, air conduction threshold was not significantly different between groups at 500, 1000, 2000, and 4000 Hz (*p* > 0.05). Post-operatively, the BAHA group (44.3 ± 14.3 and 44.5 ± 11.3) demonstrated a significantly larger hearing gain than the EACR group (20.0 ± 18.9 and 15.3 ± 19.9) in both the short and long-term periods (*p* < 0.001). Overall, the incidence of complications and need for revision surgery were comparable between groups (*p* > 0.05). Quality of life assessment revealed no statistical significance between the two groups (*p* > 0.05).

**Conclusion:** Although the quality of life and incidence of surgical complications between the two interventions was not significantly different, BAHA implantation appears to provide a better, more reliable audiologic outcome than EACR.

## Introduction

Congenital aural atresia is described as a developmental anomaly affecting the formation of the external auditory canal. It is caused by incomplete canalization through the temporal bone and is associated with an absence or under development of the tympanic membrane and ossicular chain. It affects as many as 1 in 10,000 births, with a greater incidence in males and more often occurring unilaterally (1). Any child with congenital aural atresia, either as an isolated event or found with other congenital anomalies may require intervention so that hearing and speech can develop appropriately.

The first attempt at surgical reconstruction for congenital aural atresia was performed by Kisselbach in 1883 and resulted in facial nerve paralysis and persistent hearing loss (2). Although there have been refinements in the surgery since its inception, the goal of creating an external auditory canal and middle ear system to restore hearing without the need for assistive devices has been a challenging and an elusive goal for even the most skilled surgeons. More surgeons worldwide are shifting their management paradigm for conductive hearing loss in children with congenital aural atresia to include implantation of a bone-anchored hearing aid.

Bone-anchored hearing aids were first introduced by Tjellstrom in Sweden in 1977 (3). Its main components involve the implantation of a titanium screw into the mastoid process of the skull with an abutment applied externally where the removable hearing aid may be clipped on and off (4, 5). The bone-anchored hearing aid is based on the principle of osseointegration. The titanium implant forms a direct bond to the bone itself creating a direct interface for sound to be relayed externally through the hearing aid to the skull and ultimately to the vestibulocochlear nerve (6–8).

Many studies have examined the short and long-term audiologic results and complications of each of these management options. Very few studies, however, have compared these treatments with the main purpose of determining, which is the optimal choice for children with congenital aural atresia. To date, there have been no studies that have made direct comparisons regarding quality of life. Therefore, this study compared children and adolescents with congenital aural atresia either receiving external auditory canal reconstruction (EACR) or bone-anchored hearing aid (BAHA). We measured the following outcomes – surgical complications, audiologic results, and quality of life.

## Materials and Methods

### Patient selection

The patient population was comprised of children with congenital aural atresia or stenosis who received treatment at our pediatric tertiary care center. Patients were selected from the hospital’s database if they had undergone either EACR or BAHA implantation during the time period of January 1988 to January 2011. The study was approved by the Children’s Hospital of Los Angeles Institutional Review Board.

In our institution, children are directed toward one of the treatment options primarily based on visualization of their temporal bones via CT scan and their subsequent Jahrsdoerfer score. Children with a pre-operative Jahrsdoerfer score of 6 or higher were considered as candidates for EACR. These children were offered surgery to reconstruct the external auditory canal (with or without ossicular chain reconstruction) if they could not initially be offered rehabilitation with a conventional hearing aid. Whereas children with Jahrsdoerfer scores of 5 or lower were not considered as candidates for EACR and were offered BAHA implantation. However, a discussion with the family regarding the risks and benefits of each procedure usually assisted in the management’s decision.

Data were collected retrospectively and included demographic information, such as age at surgery, sex, unilateral vs. bilateral, presence of stenosis vs. atresia, presence of microtia, association with syndrome, and side of operation.

A study of the audiometric data included the pre- and post-operative hearing tests for both treatment groups. Hearing thresholds at 500, 1000, 2000, and 4000 Hz frequencies were collected. Bone conduction, air conduction, and pure-tone average (PTA) scores were compared. Both short-term (<6 months from surgery) and long-term (>1 year from surgery) follow-up hearing assessments were collected. Hearing gain was calculated as post-operative PTA minus the pre-operative PTA.

Compiled surgical data included the procedure performed, post-operative complications, unilateral or bilateral interventions, and whether a one or two staged procedure was done in the BAHA group. Additionally, for either group, data regarding revision, including number of revisions and indication for revision were recorded. A detailed description of the atresia repair and BAHA implantation has been described elsewhere. Children were candidates for BAHA implantation if they were 5 years old or greater. When a two staged BAHA implantation was performed, the titanium implant fixture remained covered for a minimum of 6 months to allow for maximum osseointegration. In the second stage, the implant was exposed and the abutment fitted. With a single stage BAHA procedure, a 3-month-period of osseointegration was allowed before the processor was worn. Both 3 and 4 mm auto-tarauding fixtures were used in the BAHA group, but preferably a 4-mm fixture was placed when possible. For EACR group, all procedures were performed by a single senior surgeon with many years of experience in otology.

Post-operative complications for both procedures were collected. For those receiving reconstructive surgery, major complications such as canal stenosis, skin graft failure, cholesteatoma, facial nerve palsy, infection, sensorineural hearing loss, and wound dehiscence were obtained. For those patients receiving BAHA, complications such as failure of osseointegration, skin breakdown, infection, skin overgrowth, fixture loss, and others were recorded.

Patients and their families were contacted either by telephone as listed in the medical record or by pre-scheduled visits to our otolaryngology clinic at the medical center. Children who were included in the measurements of quality of life were consented for their participation prior to completion of the modified Glasgow Benefit Inventory (GBI). The GBI is a questionnaire that has been validated for quality of life measurement in otolaryngologic studies (9, 10). The quality of life issues that were addressed in this questionnaire include: general feelings toward intervention, feelings of social support, and physical benefit of intervention as outlined by daily activities, such as watching TV or talking with a group of people. The GBI used in this study has a total score of 130. The GBI is quantified with a score between −130 and 0 reflecting a diminished quality of life and a score between 0 and 130+ reflecting an improved QOL.

Statistical analyses were undertaken using IBM SPSS software (version 20.0) with significance threshold fixed at α = 5%. *t*-test analysis, one-way ANOVA, chi-square test, and Fisher’s exact test were utilized.

## Results

### Demographic data

The total number of patients enrolled in the study was 68. There were 49 subjects in the EACR group and 19 in the BAHA group. The mean age for the EACR group was 9.9 and 12.7 years in the BAHA group. The average age was significantly different between groups (*p* < 0.05). There were more males than females in both groups, but the sex distribution was not significantly different between groups (*p* > 0.05). Bilateral atresia or stenosis was found in more children in the BAHA group (84.2%) than the EACR group (38.8%) (*p* = 0.001). Bilateral interventions were performed in more children in the BAHA group (73.7%) than in the EACR group (24.5%) (*p* < 0.001). Twenty-six percent of the BAHA group had a single stage implantation. Thirty-two (65.3%) children in the EACR group and 16 (84.2%) in the BAHA group had associated microtia. Although a greater percentage of patients had microtia in the BAHA group, this result was not statistically significant (*p* > 0.05). A larger percentage of children in the BAHA group had an associated syndrome (53.3%) when compared to the EACR group (15.4%) (*p* < 0.05).

In 41 patients, we were able to collect complete audiologic data and in 44 we were able to collect follow-up information regarding surgical complications. Due to the retrospective nature of the study, the Jahrsdoerfer score was not available for the majority of patients enrolled in the study. Please refer Table 1 for more comprehensive demographic information.

**Table 1:** **Comparison of demographic information between the EACR and BAHA groups**.

Characteristic	BAHA	EACR	*p* Value
Age (year): mean ± SD (range)	12.7 ± 4.3 (7–18)	9.9 ± 4.8 (3–22)	0.03
Sex (male:female)	10:9	28:21	0.74
Side (right:left)	17:15	37:24	0.48
Associated microtia (%)	84.2	65.3	0.64
Associated syndrome (%)	53.3	15.4	0.02
Ear canal deformity (atretic:stenotic)	24:8	54:7	0.09
Bilateral intervention (%)	73.7	24.5	<0.001
Bilateral atresia or stenosis (%)	84.2	38.8	0.001

*Significance level was chosen to be α = 5%*.

### Audiologic data

#### Bone conduction

In both groups, there were no children who demonstrated any significant sensorineural hearing loss, either pre- or post-operatively. The average of pre-operative bone conduction hearing thresholds for 500, 1000, 2000, and 4000 Hz frequencies was not significantly different between the BAHA and EACR group (*p* > 0.05).

#### Air conduction

Pre-operative air conduction thresholds at all frequencies were not significantly different between the BAHA group (PTA = 64.76 ± 12.63 dB) and EACR group (PTA = 61.93 ± 9.70 dB) (*p* > 0.05) Table 2. We found that PTA significantly improved from pre-operative levels at both long and short-term hearing assessments in both EACR and BAHA groups (*p* < 0.001). But the long-term PTAs were not significantly different from short-term PTAs in either group (*p* > 0.05). Although both groups demonstrated improvement, the average hearing thresholds were significantly lower in the BAHA group compared to the EACR group for PTA and all frequencies both in short and long-term audiometric measurements (*p* < 0.001) (Tables 3 and 4).

**Table 2:** **Pre-operative hearing thresholds comparison between the BAHA and EACR groups**.

Freq	BAHA	EACR	DIF	*p* Value
500	69.76 ± 15.77	66 ± 11.36	3.76 ± 3.94	0.35
1000	68.81 ± 14.57	64.29 ± 12.01	4.52 ± 3.77	0.24
2000	62.5 ± 15.94	59.41 ± 12.66	3.09 ± 4.17	0.46
4000	59 ± 13.53	58.24 ± 12.24	0.76 ± 3.68	0.83
PTA	64.76 ± 12.63	61.93 ± 9.70	2.83 ± 3.21	0.38

**Table 3:** **Short-term post-operative hearing thresholds comparison between BAHA and EACR**.

Freq	BAHA	EACR	DIF	*p* Value
500	20 ± 8.76	42.61 ± 15.44	22.61 ± 3.89	<0.001
1000	16.88 ± 7.27	41.09 ± 20.11	24.21 ± 4.57	<0.001
2000	18.75 ± 6.71	37.82 ± 17.04	19.08 ± 3.93	<0.001
4000	24.69 ± 9.39	48.04 ± 21.15	23.36 ± 5.00	<0.001
PTA	20.08 ± 7.21	42.39 ± 16.36	22.31 ± 3.86	<0.001

**Table 4:** **Long-term post-operative hearing thresholds comparison between BAHA and EACR**.

Freq	BAHA	EACR	DIF	*p* Value
500	24.69 ± 8.65	47.42 ± 20.81	22.74 ± 4.22	<0.001
1000	19.38 ± 7.04	47.72 ± 23.82	28.35 ± 4.50	<0.001
2000	21.56 ± 8.51	40.45 ± 24.09	18.89 ± 4.70	<0.001
4000	23.13 ± 6.80	50.91 ± 25.51	27.78 ± 4.75	<0.001
PTA	22.19 ± 6.76	46.63 ± 21.30	24.44 ± 4.07	<0.001

**Figure 1:**
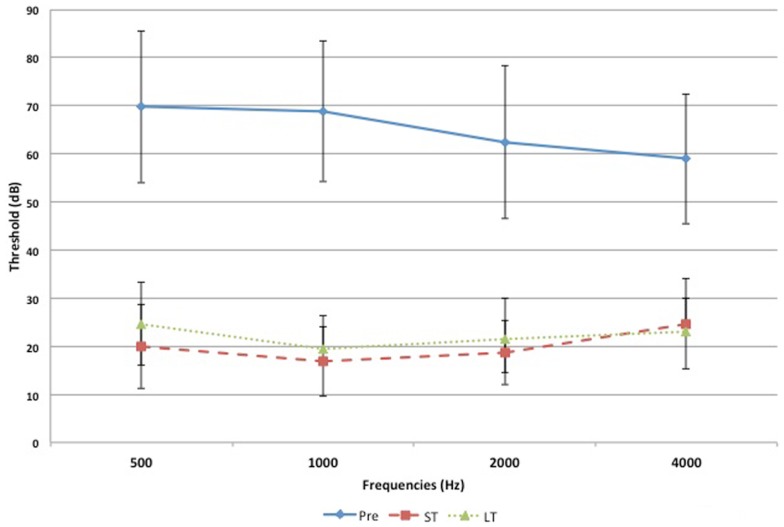
**Evolution of hearing threshold overtime for the BAHA group**.

**Figure 2:**
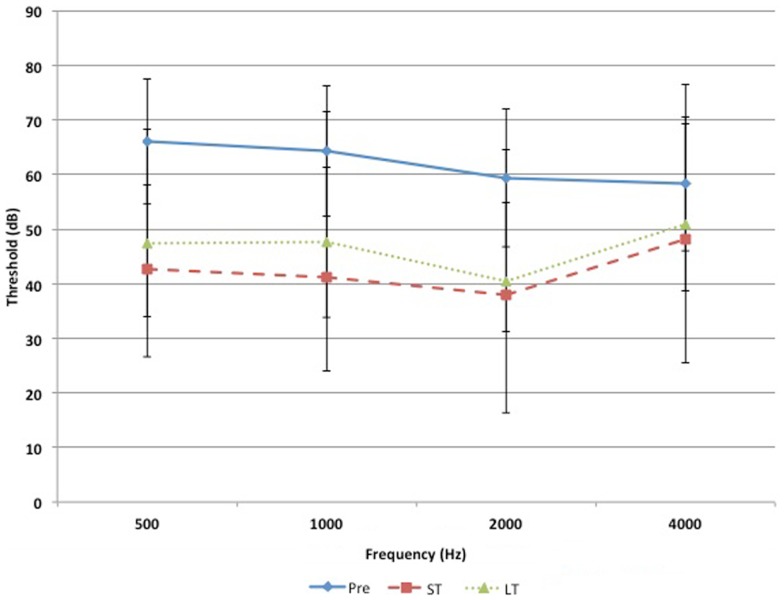
**Evolution of hearing threshold over time for EACR group**.

Figures 1 and 2 demonstrate the evolution of hearing thresholds over time. The BAHA group showed an average hearing gain of 44.3 ± 14.3 and 44.5 ± 11.3 dB in the short and long-term post-implantation periods, while the EACR group’s hearing gains were 20.0 ± 18.9 and 15.3 ± 19.9 dB at short-term and long-term, respectively. The hearing gain for the BAHA patients was significantly higher than for the EACR group for both short and long-term periods (*p* < 0.001) (Table 5). Within the EACR group, hearing gain was not significantly different between patients with or without ossicular chain reconstruction (*p*, 0.05). Associated microtia did not significantly affect the hearing gain in either groups (*p* > 0.05). A separate analysis demonstrated a greater short and long-term hearing gain for atresia than stenosis patients in both EACR and BAHA groups, although most values were not significant (Tables 6 and 7). A comparison of short and long-term PTA revealed that atresia patients had poorer outcomes than their stenotic counterparts but the difference was not significant (Table 8). A comparison between BAHA and EACR groups showed a significantly greater hearing gain both in short and long-term for atresia patients (*p* < 0.001). In stenosis subjects, hearing gain was also better with BAHA implantation than EACR but significance was reached only in the long-term (Table 9).

**Table 5:** **Comparison of short and long-term hearing gain between BAHA and EACR groups**.

	BAHA	EACR	Diff	*p* Value
ST	44.30 ± 14.34	20 ± 18.93	24.30 ± 5.27	<0.001
LT	44.53 ± 11.26	15.27 ± 19.85	29.27 ± 4.46	<0.001

**Table 6:** **Comparison of short and long-term hearing gain in EACR atresia and stenosis subjects**.

	Atresia	Stenosis	*p* Value
Hearing gain-ST	24.7 (*n* = 19)	2.3 (*n* = 5)	0.08
Hearing gain-LT	8.9 (*n* = 27)	−1.0 (*n* = 6)	0.15

**Table 7:** **Comparison of short and long-term hearing gain in BAHA atresia and stenosis subjects**.

	Atresia	Stenosis	*p* Value
Hearing gain-ST	47.6 (*n* = 13)	30.0 (*n* = 3)	0.36
Hearing gain-LT	42.9 (*n* = 14)	56.25 (*n* = 2)	0.02

**Table 8:** **Comparison of short and long-term pure-tone threshold average in EACR group**.

	Atresia	Stenosis	*p* Value
PTA-ST	38.5 (*n* = 19)	56.3 (*n* = 5)	0.15
PTA-LT	42.8 (*n* = 27)	64.0 (*n* = 66)	0.13

**Table 9:** **Comparison of short and long-term hearing gain in atresia and stenosis subjects**.

	EACR	BAHA	P
**ATRESIA**			
ST hearing gain [mean (*n*)]	24.7 (*n* = 19)	47.6 (*n* = 13)	<0.001
LT hearing gain [mean (*n*)]	18.9 (270)	42.9 (*n* = 14)	<0.001
**STENOSIS**			
ST hearing gain [mean (*n*)]	2.3 (*n* = 5)	30 (*n* = 3)	>0.05
LT hearing gain [mean (*n*)]	−1.0 (*n* = 6)	52.3 (*n* = 2)	<0.05

Subsequent hearing aid use was required in 9 (34.6%) who underwent EACR. Four children used a soft-band BAHA and three used conventional hearing aids post-operatively. Two children underwent BAHA implantation after EACR.

The median short and long-term follow-up times for the BAHA group were 3.5 and 18 months and for the EACR group were 5 and 24 months.

#### Complications

The total number of complications in each group were comparable with 55.5% occurring in the BAHA group and 69.2% in the EACR group. In the EACR group, there was a need for revision surgery (30.8%), canal restenosis (30.8%), skin graft failure (7.7%), cholesteatoma formation (7.7%), wound infection (42.3%), and tympanic membrane lateralization or rupture (7.7%). In the BAHA group, we found the need for revision surgery (11.1%), skin overgrowth (33.3%), localized infection around the implant (50.0%), and fixture loss (5.6%). Neither the number of revision surgeries nor the total number of complications were significantly different between the two groups (*p* < 0.05).

#### Quality of life

Of the total patients enrolled in the study, only 23 were contacted and consented for participation in the quality of life survey. The mean age of the patient at the time of questionnaire was 17.3 years. Of these patients, 65.2% had EACR and 34.8% received a BAHA. Scoring was done manually, with the best outcome graded as a 5/5, no change as a 3/5, and the worst outcome as a 1/5. Scores were added, and a higher score indicated a better quality of life with the given intervention. The total mean score for the atresia surgery group was 93.7 and for the BAHA group was 98.9 with no statistical significance demonstrated (*p* = 0.25).

## Discussion

Congenital aural atresia is a rare condition affecting 1 in 10,000–20,000 children and is typically sporadic, but may be associated with other craniofacial anomalies (11–13). Interventions are required at an early age, especially if the condition is bilateral, as it will affect the child’s speech and language development.

The first attempt at surgical correction of congenital aural atresia occurred over 150 years ago. Over the last three decades, multiple refinements of the technique have been described and surgical results have been reported. The procedure continues to involve the primary surgical steps of canaloplasty, tympanoplasty, with or without ossicular chain reconstruction, and skin grafting of the external ear canal (14). Although simple in description, the goal of creating a patent external auditory canal and an ossicular chain that allows for functional hearing is a challenging goal for even the most skilled surgeons. Frequent surgical complications have been encountered and include: recurrent canal stenosis, recurrent otitis externa, cholesteatoma, facial nerve paralysis, poor cosmesis, and variable hearing outcomes. There have been also reports of rare complications such as salivary fistula and middle ear cholesteatoma (15). Additionally, failures to achieve normal hearing may still require an external hearing aid. Although quality of life has not been studied extensively in patients who have undergone EACR, at least one adult study indicated that only about 25% reported benefit after the procedure (16, 17).

Bone-anchored hearing aids have been a more recent development in treating children with congenital aural atresia, especially in those children whose anatomy makes reconstruction impossible (18). The bone-anchored hearing aid system bypasses the external auditory canal and middle ear and allows for sound to be transmitted directly to the cochlea through a hearing aid device that is connected to an implant placed into the mastoid process. Overall, the BAHA system has been shown to be reliable and effective in children and adults with both congenital and acquired conductive hearing deficits (19–22). The procedure to perform implantation of the BAHA system is not complicated. Children were eligible for implantation if they were at least 5 years old. However, in children because of their thinner calvarial bone, the implantation is often carried out through a two stage technique in order to allow for maximum osseointegration. The first procedure involves placement of the implant into the cranial bone and the second is performed 6 months later to allow exposure of the implant and maturation of the surrounding skin and soft tissue. The complications associated with BAHAs include: infection and thickening of the skin around the implant site, and failed osseointegration or fixture loss (4, 23–26). Audiologic data and subsequent impact on quality of life with the BAHA have been promising. Evaluations of children’s audiograms after being fitted for a BAHA show that 85% of children can achieve air-bone gap closure (26). Several studies measuring quality of life after BAHA implantation demonstrate an improvement in health and hearing in several different situations (4, 26–28). However, some patients have been frustrated with the BAHA due to wound problems and fixation failures (29).

This study was a comparison of children and adolescents with congenital aural atresia who received either EACR or BAHA. To date, there have been few studies which directly compared the two management options. Overall, the surgical complications and audiologic results appear to be consistent with what has been previously reported with some exceptions.

We did not find marked demographic differences when comparing the two groups pre-operatively except for age at surgery, the presence of an associated syndrome, and bilaterality of atresia or stenosis, which were all significantly greater in the BAHA group. Many of these observations can be explained by our selection process for each procedure, which was primarily based on the Jahrsdoerfer classification scale. It has been well noted that many children with specific syndromes (i.e., Treacher Collins and Goldenhar), have bilateral microtia and atresia, have lower Jahrsdoerfer scores and are often poor candidates for atresia repair (30). Therefore, our selection algorithm would naturally place a greater number of children with these conditions in the BAHA group. Unfortunately, because of the retrospective nature of the study, we were unable to collect information regarding Jahrsdoerfer scores for many of our patients and so, we cannot confirm this assumption, nor can we comment about how the score and severity may have affected our outcomes. The greater age of our BAHA patients may be attributed to the strict age criterion (5 years or older) used before surgical implantation would be considered. Our BAHA group may also be older because many of these children had associated syndromes, which because of their complex natures may have led to other procedures having priority before BAHA implantation.

Children in both groups had similar pre-operative air conduction thresholds and this finding differs from that reported by Bouhabel et al. who demonstrated a worse pre-operative audiologic status in their BAHA group (31). In contrast to their study wherein children predominantly with external auditory canal stenosis were reconstructed, we may have had a larger number of EACR patients with complete atresia and consequently poorer pre-operative hearing thresholds.

Post-operatively, we found that patients in the EACR group had poorer audiologic outcomes when compared to the BAHA group. We also found a mild deterioration over time in the hearing gain of EACR group when evaluating short and long-term outcomes. This is somewhat similar to findings presented by others. Evans et al. and Chang et al. reported an average hearing gain of 17.3 and 20 dB respectively (32, 33). Moreover, Chang et al. also found diminishment in his audiologic thresholds over time, finding a greater decline in hearing specifically in revision cases. He also noted that the severity of microtia appeared to predict the long-term hearing status with a higher grade microtia correlating with a worse audiologic outcome (33). In comparison, our evaluation of hearing gain in the EACR group did not reveal any significant difference when analyzing for the variables of microtia or atresia vs. stenosis. Again, it is possible that our results differ because we lack information regarding the severity of microtia and Jahrsdoerfer classification scores. There seemed to be only a few patients in the EACR group, who appeared to warrant or feel the need for a hearing aid post-operatively and this may be due to the fact that many children may have had normal hearing in the contralateral ear. Other studies have reported that up to 90% of patients may require some form of amplification after EACR (32, 34).

Children who underwent BAHA implantation had significant improvement in their hearing post-operatively in both the short and long-term. Their mean hearing gain at 6 months and at 1 year remained stable over time. These results are similar to outcomes reported by other authors. Further analysis did not reveal significant differences when atresia and stenosis patients were compared, either within or between EACR and BAHA groups. This is likely due to the small number of stenotic patients included in the study.

Overall, our incidence of complications is also compatible with what has been documented in the literature. There was no significant difference in the total number of complications between groups. We did have a high occurrence of canal restenosis in the EACR group (30.8%), but this is still in accordance with reports by other authors who have noted an incidence of 8–29% (35, 36). Some authors have identified that young age and a severity of microtia can be related to a higher incidence of post-operative canal restenosis (33, 37, 38). Because we did have a large number of young children with microtia in our EACR group, it is possible that these factors may have contributed to our higher restenosis rate. Our rate of fixture loss in the BAHA group (5.6%) appears similar to the 5–29% incidence rate that has been reported previously (39). The single fixture loss that occurred was not trauma related and occurred in a 10-year-old child after several months of wearing his processor. Fixture loss has been reported to be related to the age of the patient, occurring with greater frequency in children <5 years and if a 3-mm fixture was used (40). Our low rate of fixture loss may be related to performing BAHA strictly in children 5 years of age or greater and our preference for 4 mm fixtures when possible. In general, results regarding skin infection or skin overgrowth in BAHA patients are fraught with variability due to a lack of standardization and objectivity in reporting. Skin complications in some series have been reported to range from 22 to 78% (39, 41). Our incidence of skin infections (50%) and skin overgrowth (33.3%) appear comparable to prior studies. Generally, these were minor complications which were self-limiting and easily treated with topical medications or oral antibiotics. Revision surgery occurred in fewer BAHA patients (11.1%) than EACR (30.8%), but this difference was not statistically significant. In the EACR group, the majority of patients underwent revision surgery for canal restenosis, while in the BAHA group, revision surgery was required primarily for complete skin overgrowth. Our rate of revision surgery for both groups appears to be similar to that found by other authors with rates of between 15 and 26.6% in EACR patients and 8 and 44% BAHA patients (31, 41).

The overall quality of life measurements indicated that children in both groups derived benefit from each of the interventions. We found no significant difference between groups in the GBI scores. We are unaware of any other publications which have compared quality of life between BAHA and EACR. However, it should be noted that there may be several limitations related to this portion of the study. Most importantly, the small number of subjects that underwent questionnaire administration may have influenced our results to some degree. Furthermore, there may have been a great deal of lag time between surgical intervention and questionnaire administration for many patients. This discrepancy may have led to some recall bias. Because some younger subjects may have required assistance to answer their questionnaires by proxies, this may have also compromised results as well. We also did not focus on whether a subject received unilateral or bilateral surgical interventions and this may have been an important factor in quality of life measurements for both groups.

## Conclusion

Although the quality of life between the two interventions was not significant, BAHA implantation appears to provide a better, more reliable audiologic outcome than EACR. Surgical complication rates and need for revision surgery were equivocal between the two groups. Because our findings failed to find significant differences in all of our measured outcomes, there does not appear to be one intervention that is visibly the optimal preference for children with congenital aural atresia. For this reason, at our institution we will continue to use our current decision making paradigm to manage patients with congenital aural atresia. Further prospective studies may assist in determining if a sub-population of children may fare better with one technique or the other.

## Conflict of Interest Statement

The authors declare that the research was conducted in the absence of any commercial or financial relationships that could be construed as a potential conflict of interest.
